# Replication forks associated with long nuclear actin filaments in mild stress conditions display increased dynamics

**DOI:** 10.17912/micropub.biology.001259

**Published:** 2024-07-31

**Authors:** Chiara Merigliano, Maria Dilia Palumbieri, Massimo Lopes, Irene Chiolo

**Affiliations:** 1 Molecular and Computational Biology Department, University of Southern California, Los Angeles, United States; 2 Institute of Molecular Cancer Research, University of Zurich, Zurich, Switzerland

## Abstract

Nuclear actin filaments (F-actin) form during S-phase and in response to replication stress to promote fork remodeling and repair. In mild replication stress conditions, nuclear actin polymerization is required to limit PrimPol recruitment to the fork while promoting fork reversal. Both short and long filaments form during this response, but their function in the nuclear dynamics of replication sites was unclear. Here, we show that replication centers associated with long nuclear actin filaments become more mobile than the rest of the forks, suggesting relocalization of replication sites as a response to prolonged fork stalling and/or fork breakage, even in response to mild replication stress.

**Figure 1. Replication sites associated with nuclear F-actin acquire mobility.**
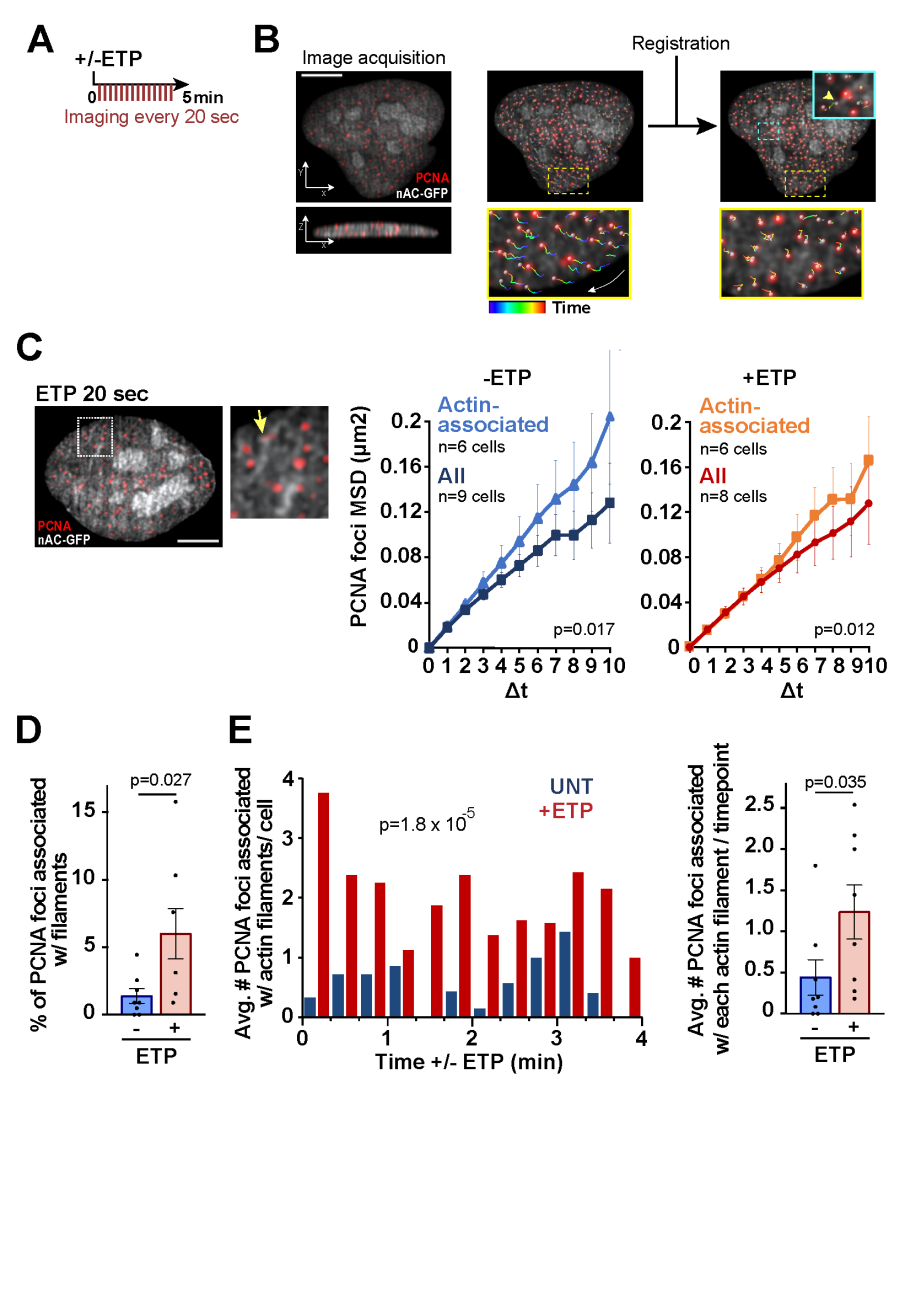
A: Experimental setup. B: Example of image acquisition and registration of a human U20S cell in S-phase, expressing PCNA-CB-RFP and nAC-GFP. Left image: top and side view of the cell. Middle and right images show the same cell before and after registration. Zoomed details in yellow squares show examples of tracks corrected by the registration. The blue square shows an example of track of a PCNA focus (yellow arrowhead) associated with an actin filament. C: Left: Representative image of another U20S cell in S-phase, with zoomed detail highlighting examples of nuclear actin filaments and a PCNA focus colocalizing with a filament (yellow arrow). Right: MSD analysis of PCNA foci in cells expressing nAC-GFP and transiently transfected with PCNA-CB-RFP, without (-) or after (+) treatment with Etoposide (ETP). For actin-associated PCNA foci: N= 24 foci for -ETP and N=26 foci for +ETP. For all PCNA foci: N=2376 foci for -ETP; N=2105 foci for +ETP. Data are mean ± SEM, from 3 or more independent experiments. Data for all PCNA foci are from (Palumbieri et al. 2023). p-values were calculated with extra sum-of-squares F-test, nonlinear regression for curve fitting. Δt, time intervals (intervals were 20 sec each). D: Quantification of the number of PCNA foci associated with F-actin in untreated cells (-) or after (-) ETP treatment. p-value calculated with two-tailed T test. E: Quantification of the average number of PCNA foci associated with each filament for each time point or quantification of the percentage of PCNA foci associated with filaments relative to all PCNA foci. Data are mean ± SEM. p-value calculated with two-tailed Mann–Whitney test. Scale bar = 5 μm.

## Description

Nuclear actin filaments (F-actin) are central regulators of the DNA damage response, which promote the dynamics of repair foci to facilitate their repositioning in the nucleus and repair (Caridi et al. 2019, Rawal et al. 2019) . For example, long nuclear actin filaments assemble in response to heterochromatic double-strand breaks (DSBs), which work in concert with nuclear myosins to relocalize repair sites from the heterochromatin domain to the nuclear periphery in *Drosophila* cells (Chiolo et al., 2011, Ryu et al. 2015, Caridi et al. 2018a, Dialynas et al. 2019, Merigliano et al. 2024) , and this response is needed to prevent ectopic recombination while promoting “safe” homologous recombination (HR) repair of repeated sequences (Ryu et al. 2015, Caridi et al. 2018a, Dialynas et al. 2019, Merigliano et al. 2024) (reviewed in Merigliano and Chiolo 2021, Rawal et al. 2019). A similar response has been shown in mouse cells, suggesting conserved pathways (Caridi et al. 2018a). F-actin also assembles in response to DSBs to facilitate focus clustering and resection during HR repair in human and *Drosophila* cells (Caridi et al. 2018a, Schrank et al. 2018, Zagelbaum et al. 2023) , and to facilitate the formation of DSB-capturing nuclear envelope tubules (dsbNETs) that promote DSB repair in human cells (Shokrollahi et al. 2024) .

Several studies also established the importance of F-actin and myosins in replication fork remodeling and repair (Lamm et al. 2020, Pinzaru et al. 2020, Han et al. 2022, Nieminuszczy et al. 2023, Palumbieri et al. 2023, Shi et al. 2023) (reviewed in (Wollscheid and Ulrich 2023, Ulferts et al. 2024) ). Specifically, prolonged treatment with APH or HU induces the formation of bright and persistent nuclear actin filaments (Belin et al. 2015, Lamm et al. 2020) that mobilize stalled forks marked by FANCD2, induce the directed motion of replication sites along the filaments, and promote fork repair (Lamm et al. 2020) . Inhibition of Myosin II affects these dynamics, consistent with a role for myosins in these movements (Lamm et al. 2020) .

In addition, short treatments with low dose of etoposide (ETP) or camptothecin (CPT) that induce mild replication stress largely without fork breakage trigger the formation of both long and short nuclear actin filaments immediately after treatments (Palumbieri et al. 2023) . In these conditions, F-actin polymerization limits PrimPol-mediated re-priming, thereby promoting fork reversal and preventing DNA gap formation during replication (Palumbieri et al. 2023) . Misregulation of this pathway results in chromosome abnormalities (Palumbieri et al. 2023) , revealing its importance to genome integrity. Interestingly, ~90% of these filaments assemble short foci and patches in fixed cells, while only ~10% form longer structures (length > 2.5 um (Palumbieri et al. 2023) ). Given that fork reversal is a global response to replication stress, which even extends to undamaged forks (Mutreja et al. 2018) , short actin filaments might be the main structures responsible for regulating the balance between PrimPol activity and fork reversal (Palumbieri et al. 2023) . The function of long actin filaments in this response is unclear, and whether these structures are linked to increased nuclear dynamics of a subset of replication sites remains unknown.

Here, we addressed these questions by investigating the dynamics of replication sites (PCNA foci) associated with long nuclear actin filaments in mild replication stress conditions induced by ETP. We used U2OS cells expressing fluorescently-tagged PCNA chromobody (PCNA-CB-RFP) to label replication sites and a NLS-actin-chromobody (nAC-GFP) to label nuclear F-actin. Of note, nAC mostly detects filaments characterized by longer structure and higher intensity (Caridi et al. 2018a, See et al. 2021, Palumbieri et al. 2023) . These filaments are typically characterized by high dynamics and fast disassembly, suggesting their transient nature (Palumbieri et al. 2023) .

Cells were treated with or without 200 nM ETP and imaged every 20 sec for 5 min ( Figure 1A ). After image acquisition, small translational and rotational movements were corrected ("registration") using PCNA foci as a reference ( Figure 1B ). PCNA foci were then tracked a second time post-registration to establish their dynamics in the nuclei (see Methods). A mean square displacement (MSD) analysis was performed on each PCNA focus trajectory and average MSD values were calculated ( Figure 1C ) (Caridi et al. 2018a, 2018b, and Methods). As previously shown, most PCNA foci display similar mobility before and after mild ETP treatment ( Figure 1C and Palumbieri et al . 2023), though a significant fraction of forks undergoes remodeling in response to ETP (Palumbieri et al. 2023) . These data suggest that remodeling of stressed forks does not require extensive mobilization of these sites.

Next, we specifically examined the dynamics of PCNA foci detectably associated with F-actin, which were conservatively defined as PCNA foci colocalizing with nAC filaments by live imaging ( Figure 1C ). These correspond to 1-6% of total PCNA foci in -/+ ETP conditions ( Figure 1D ), and this low percentage might reflect either a low number of forks involved or very transient events associated with a larger number of forks.

We extracted the positional data from the trajectories of F-actin-associated PCNA foci and calculated the corresponding MSD values. The average MSD curves for PCNA foci associated with nuclear F-actin are significantly higher than those calculated for all PCNA signals ( Figure 1C ), revealing more movement. These values are similar for cells treated or not treated with ETP ( Figure 1C ). However, the number of PCNA foci associated with nuclear F-actin increases upon stress induction ( Figure 1D,E ). This results not only from a higher number of filaments present in response to ETP (Palumbieri et al. 2023) , but also from a higher number of PCNA foci associated with each filament ( Figure 1E ), while filament length remains constant between cells untreated and treated with ETP (Palumbieri et al. 2023) .

We conclude that the small fraction of PCNA signals colocalizing with nuclear F-actin at each time point display increased mobility even during unperturbed replication. In light of the functional role of actin filaments in DNA repair (Nagai et al. 2008, Belin et al. 2015, Aymard et al. 2017, Caridi et al. 2018a, Schrank et al. 2018, Dialynas et al. 2019, Shokrollahi et al. 2024) , we suggest that PCNA foci with increased mobility in untreated conditions correspond to a few forks undergoing persistent stalling or breakage at difficult-to-replicate regions. Mild replication stress increases fork stalling and F-actin assembly, resulting in a higher fraction of mobilized forks.

Previous studies showed that stalled or broken forks relocalize to the nuclear periphery for repair (Su et al. 2015, Kramarz et al. 2020, Lamm et al. 2020, Pinzaru et al. 2020, Whalen et al. 2020) . While the movement we observe is relatively limited ( Figure 1B, arrow ), it is compatible with foci reaching the nuclear periphery given the low thickness of these cells ( Figure 1B ).

Together, these results support a model where the few replication forks that permanently stall or break during normal replication or in clinically relevant low-dose of cytotoxic treatments are mobilized for repair through the formation of long, transient, and dynamic nuclear actin filaments, and this mobilization likely contributes to chromosome stability by promoting fork relocalization, repair, and restart.

## Methods

**Cell cultures**

Human osteosarcoma U2OS cells were cultured in DMEM (41966-029, Life Technologies) supplemented with 10% fetal bovine serum (FBS, GIBCO), 100 U/ml penicillin and 100mg/ml streptomycin in an atmosphere containing 6% CO2 at 37°C. The PCNA chromobody (ChromoTek) was transiently transfected in U2OS cells stably expressing nAC-GFP (kind gift from R. Grosse) with Lipofectamine 3000 (ThermoFisher Scientific) according to manufacturer’s instructions, 24 h before imaging.

**Live cell imaging**

U2OS cells expressing PCNA-CB-RFP and nAC-GFP were seeded on poly-L-lysine-coated microslides (Ibidi). The culture medium was replaced with colourless DMEM (FluoroBrite DMEM, ThermoFisher Scientific) 2 h before imaging. Imaging was performed using an incubation system for live cell imaging (cellVIVO) at 37°C and 5% CO2 on an Olympus IXplore SpinSR10 with a Yokogawa CSU-SoRa disk and UPLSAPO 100×/1.3 NA oil-immersion objective. Images were acquired every 20 s for about 5 min. Z stacks were collected with 0.3 μm intervals over a 12 μm range. 200 nM ETP or H2O (NT) were added to the cells just before imaging.

**Cell registration and tracking of PCNA foci**

We utilized Imaris (v. 9.5) to correct (“registration”) for minor translational and rotational motions of the cell/nucleus during the experiment and to track foci for motion analysis, using techniques similar to those previously described for DNA damage foci (Caridi et al. 2018a, 2018b, See et al. 2021) .

First, cells were registered by automatically detecting and tracking all PCNA foci and by correcting rotational and translational cell drift using these foci as a reference ( Figure 1B ). Automated PCNA focus tracking was facilitated by reducing background signals and small focus vibrations along the Z-stack using Imaris, as follows. Background signals were reduced by applying the “Baseline Subtraction” option in the “Thresholding” function of “Image Processing” to the channel with PCNA foci. In addition, vibrations along the Z-stack were minimized by applying the “Smooth Time” function of “Image Processing” with a filter width of 1 to all time points (see also (Caridi et al. 2018b)). PCNA foci were detected using the "Spot Detection Tool” with 0.65 um particle size. Tracks were built using the algorithm for “Autoregressive Motion”, with parameters “Max Distance" set at 1 μm, "Max Gap Size" set at 3, and using the “filling the gaps with all detected objects” option in Imaris.

Second, PCNA foci were re-tracked after registration. Tracking of PCNA foci was done similarly to the registration process, except that a new “spot” was generated for each tracked focus. Focus positional data were then extracted as .cvs files for MSD analyses, from the “Statistics" tab. For multiple foci, the .cvs file contains all the positional data of each focus.

**MSD analyses**

Positional data in excel were analyzed in Matlab (MathWorks) to calculate MSDs as previously described (Caridi et al. 2018a, Caridi et al. 2018b, Miné-Hattab and Chiolo 2020, See et al. 2021) . Given the high number of PCNA foci (about 200/300 per cell, Figure 1B,C ) that needed to be processed with previously published scripts (Caridi et al. 2018a), we generated a new script to automate the file conversion (Extended Data Script 1).

**Statistical analysis**

Statistical analyses were performed using Prism6 (Graphpad Software).

## Reagents

**Table:** 

**Chemicals**		
**Name**	**Source**	**catalogue number**
Etoposide	Sigma-Aldrich	E1383
DMEM	Thermo Fisher Scientific	41966-029
FluoroBrite DMEM	Thermo Fisher Scientific	A1896701
Pen/Strep	Thermo Fisher Scientific	11548876
Lipofectamine 3000	Thermo Fisher Scientific	L3000008
**Plasmids**		
**Name**	**Source**	**catalogue number**
PCNA-CB-RFP	Chromotek	ccr
NLS–GFP–actin chromobody	Chromotek	acgn
**Software and Algorithms**		
**Name**	**Source**	**catalogue number**
IMARIS image analysis software version 9.5	Bitplane	https://imaris.oxinst.com/packages
Matlab	MathWorks Inc.	https://www.mathworks.com
GraphPad Prism 6	GraphPad	www.graphpad.com

## Extended Data

Description: Extended data script 1. This script enables automated pre-processing of focus positional data for MSD analyses. The input is an Excel file extracted from Imaris (‘Detailed’.csv), and re-ordered by track ID. In this file, the positional data for each focus are shown for subsequent time points, and followed by the next focus. The script creates separate files, one for each focus, already formatted for MSD analyses, i.e. with time points starting from 001 in the first column and XYZ coordinates in the 3 subsequent columns. The resulting set of files is entered as is a previously published script for MSD calculation (Caridi et al. 2018b).. Resource Type: Text. DOI: 10.22002/x5gxk-2xk32
